# 非手性-手性色谱-预测多反应监测法分析中药毛前胡的化学成分

**DOI:** 10.3724/SP.J.1123.2020.08024

**Published:** 2021-06-08

**Authors:** Xia XU, Ting LI, Jinru JIA, Huiting TANG, Jun LI, Yunfang ZHAO, Yuelin SONG

**Affiliations:** 北京中医药大学, 中药学院中药现代研究中心, 北京 100029; Modern Research Center for Traditional Chinese Medicine, School of Chinese Materia Medica, Beijing University of Chinese Medicine, Beijing 100029, China; 北京中医药大学, 中药学院中药现代研究中心, 北京 100029; Modern Research Center for Traditional Chinese Medicine, School of Chinese Materia Medica, Beijing University of Chinese Medicine, Beijing 100029, China; 北京中医药大学, 中药学院中药现代研究中心, 北京 100029; Modern Research Center for Traditional Chinese Medicine, School of Chinese Materia Medica, Beijing University of Chinese Medicine, Beijing 100029, China; 北京中医药大学, 中药学院中药现代研究中心, 北京 100029; Modern Research Center for Traditional Chinese Medicine, School of Chinese Materia Medica, Beijing University of Chinese Medicine, Beijing 100029, China; 北京中医药大学, 中药学院中药现代研究中心, 北京 100029; Modern Research Center for Traditional Chinese Medicine, School of Chinese Materia Medica, Beijing University of Chinese Medicine, Beijing 100029, China; 北京中医药大学, 中药学院中药现代研究中心, 北京 100029; Modern Research Center for Traditional Chinese Medicine, School of Chinese Materia Medica, Beijing University of Chinese Medicine, Beijing 100029, China; 北京中医药大学, 中药学院中药现代研究中心, 北京 100029; Modern Research Center for Traditional Chinese Medicine, School of Chinese Materia Medica, Beijing University of Chinese Medicine, Beijing 100029, China

**Keywords:** 非手性-手性色谱, 预测多反应监测, 对映异构体拆分, 角型吡喃香豆素, 毛前胡, achiral-chiral liquid chromatography (achiral-chiral LC), predictive multiple reaction monitoring (predictive MRM), enantioseparation, angular-type pyranocoumarins (APs), Ligustici Radix

## Abstract

中药毛前胡为伞形科植物短片藁本*Ligusticum brachylobum* Franch.的干燥根,主要用于治疗风热咳嗽痰多、痰热喘满、咯痰黄稠等证,富含香豆素类化学成分,含有多组对映异构体和非对映异构体。为了深入研究毛前胡的化学成分组成,特别是对映异构体的组成,研究建立了非手性-手性色谱-预测多反应监测法(achiral-chiral-LC predictive MRM),同步实现毛前胡化学成分的化学选择性和立体选择性分离,以及高灵敏度定性分析。非手性色谱和手性液相色谱-串联质谱系统结合了RP-C_18_色谱柱的高效化学选择性分离能力以及手性色谱柱的立体选择性优势,有效避免了中心切割非手性-手性二维液相色谱构造复杂、重现性难以满足定量要求等缺陷。采用小内径核-壳型RP-C_18_色谱柱作为前端化学分离柱,实现结构类似香豆素的高效化学选择性分离;采用反相大内径AD-RH手性色谱柱,实现对映异构体的手性拆分;采用预测多反应监测模式,实现化学成分的高灵敏度检出;利用增强子离子扫描模式(EPI)采集各色谱峰的二级质谱信息,鉴定化学结构。通过定量离子对、定性离子对及两者的比值,判定是否为对映异构体。利用所构建的非手性-手性色谱耦联系统从毛前胡中共鉴定出60个化学成分,其中8对香豆素对映异构体得到了良好分离。本研究为毛前胡以及含有对映异构体中药的深入定性、定量分析提供可靠的方法。

## Abstract (en)

Ligustici Radix (Chinese name: maoqianhu) consists of the dried roots of *Ligusticum brachylobum* Franch., which is mainly distributed in the Yunnan and Sichuan provinces. This herbal medicine has been primarily used for the treatment of cough in traditional Chinese medicine. Ligustici Radix is rich in coumarin derivatives. Interestingly, enantiomers and diastereomers are widely used for these coumarins, thus posing a great challenge for in-depth chemical profile characterization. In the present study, a new analytical platform, achiral-chiral liquid chromatography-tandem mass spectrometry (achiral-chiral LC-MS/MS) was configured to profile the chemical composition of Ligustici Radix. Because achiral and chiral columns were serially coupled, especially enantiomers, both chemically and enantiomerically selective separations could be accomplished simultaneously. The newly configured achiral-chiral LC-MS/MS platform did not require any electronic valve; hence, it could overcome the drawbacks of heart-cutting achiral-chiral two-dimensional LC, i. e., sophisticated instrumentation and limited reproducibility due to the use of electronic valve(s) and the undesired retention time shift across different analytical runs. Some available candidates for chemically selective or enantiomerically selective separation were assayed; then, Capcell core RP-C_18_ column that was packed with core-shell type particles, and AD-RH column embedding amylose coated particles were employed the achiral and the chiral columns, respectively. The narrow-bore core-shell RP-C_18_ column served as the front tool to achieve efficient chemoselective separation of coumarin analogs, and enantioselective enantiomers were obtained by using a wide-bore AD-RH chiral column. The predictive multiple reaction monitoring (predictive MRM) mode allowed for the sensitive detection of potential components, and an enhanced product ion (EPI) scan, which was a unique function of Qtrap-MS, was programmed to record the MS^2^ spectra for all captured signals and thus aid structural annotation. Online energy-resolved mass spectrometry (online ER-MS) was introduced to pursue the suitable collision energy for each compound; in particular, inferior collision energy instead of the optimal one was utilized to suppress the response of the primary components such as praeruptorin A, B and pteryxin. The criteria to judge enantiomers or not included identical quantitative and qualitative precursor-to-product ion transitions, identical quantitative versus qualitative responses, and longer retention times from achiral-chiral LC over single-column achiral LC. As a result, a total of sixty components were observed and structurally identified. In particular, enantiomerically selective separations were achieved for eight enantiomers, *cis*-khellactone (CKL), qianhucoumarin G (QC-G), pteryxin (Pte), praeruptorin A (PA), *cis*-3'-isovaleryl-4'-acetylkhellactone (IAK), praeruptorin B (PB), praeruptorin E (PE), and *cis*-3',4'-diisovalerylkhellactone (DIK). Notably, none of the enantiomers were present as racemates; instead, the proportion of one enantiomer in each pair was greater than the other. Achiral-chiral LC-predictive MRM is a feasible choice for the quantitative and qualitative analyses of Ligustici Radix as well as other herbal medicines characterized by enantiomers and diastereomers.

中药毛前胡(Ligustici Radix)为伞形科植物短片藁本(*Ligusticum brachylobum* Franch.)的干燥根,其性温,味甘、辛,入肝、脾、膀胱三经,主要用于治疗风热咳嗽痰多、痰热喘满、咯痰黄稠等证^[1]^。毛前胡植物形状、功能主治与中药前胡(Peucedani Radix)均相似,常被用作前胡的替代品,但因其根茎部密布较为坚硬的残存叶鞘纤维,难以除净,故药材称为毛前胡。国内外对其化学成分研究较少,制约了毛前胡的深度开发。因此,本研究对其化学成分进行深入阐明,以期为其进一步开发利用提供理论依据,同时也为其他伞形科中药中香豆素类成分分析提供借鉴。利用课题组前期^[2,3,4]^建立的中心切割非手性-手性二维液相色谱-串联质谱系统(heart-cutting achiral-chiral 2D LC-MS/MS)以及直接进样-三维质谱法(DI-3D MS)对前胡和毛前胡的化学成分进行了系统的对比分析,通过比较各信号峰的多级质谱数据和保留时间,发现毛前胡的主要化学成分与前胡类似,均以顺式角型吡喃香豆素(即为顺式凯琳内酯衍生物)为主,且含有多组对映异构体。对前胡而言,多对对映异构体之间的含量差异性大,表现为对映异构体过量的现象^[5]^,并且对映异构体间往往呈现出药理活性^[6]^和代谢^[7]^差异。我们推测毛前胡中的这些对映异构体也并非以外消旋体的形式存在,而是其中一种构型含量较高,通常称为优映体。因此,在对中药毛前胡进行化学成分深入分析时,应对其对映异构体进行立体选择性分离,明确对映体之间的含量比例,从而更好地实现质量控制。

近年来,一些新兴的色谱填料技术例如核-壳颗粒^[8]^、亚微米颗粒^[9,10]^和整体柱^[11,12]^,使得LC的分离能力得到了显著提高,尤其是核-壳色谱柱因其具有更高的柱效、更低的反压以及更高的分析效率,为中药中结构类似物和非对映异构体的化学选择性分离提供了保障。手性色谱柱快速发展,多种涂敷型、键合型手性色谱填料^[13]^的不断涌现使得绝大多数对映异构体都可以实现立体选择性分离。然而,手性色谱柱往往具有较差的化学选择性分离能力^[13]^,而RP-C_18_色谱柱虽然具有高效的化学选择性分离能力,但无法实现对映异构体的手性分离。

本研究将反相非手性色谱柱和手性色谱柱通过聚醚醚酮管线在线连接,该系统结合了RP-C_18_色谱柱的高效化学选择性分离能力和手性色谱柱的立体选择性优势,有效避免中心切割非手性-手性二维液相色谱-质谱联用系统的缺点,以期同步实现中药毛前胡化学成分的化学选择性和立体选择性分离。同时,充分利用类似中药(如前胡等)化学成分研究成果^[2]^,进而采用三重四极杆质谱特有的预测多反应监测(predictive MRM)模式^[14,15,16]^实现化学成分的高灵敏度、高选择性分析。利用该系统从中药毛前胡中鉴定了其中60个化学成分,包括4个氨基酸类(AAs)、43个角型吡喃香豆素类(APs)、10个线型呋喃香豆素类(LFs)、2个线型吡喃香豆素类(LPs)以及1个简单香豆素类(SC)化合物。

## 1 实验部分

### 1.1 仪器、试剂与材料

U-3000双三元色谱仪,配备两个三元泵(即左泵和右泵)、自动进样器、柱温箱和紫外检测器(美国Thermo Fisher公司); SCIEX 5500 Qtrap质谱仪(美国Sciex公司); Milli-Q超纯水净化系统(美国Millipore公司); Mettler ME204型电子分析天平(瑞士Mettler Toledo公司);超声波清洗器(南京垒君达超声电子设备有限公司); Premixer Assy混合器(日本Shimadzu公司)。

色谱级和质谱级甲醇、乙腈以及质谱级甲酸均购自美国Thermo Fisher公司;实验用水由Milli-Q纯水系统制备;其余试剂均为分析纯,由北京化工厂提供。中药毛前胡购于北京同仁堂,经北京大学屠鹏飞教授鉴定为伞形科植物短片藁本*Ligusticum brachylobum* Franch.的干燥根。标本存放于北京中医药大学中药学院中药现代研究中心。

### 1.2 实验方法

1.2.1 样品前处理

取干燥的毛前胡药材,粉碎机粉碎,过20目筛。精密称取1.0 g粉末样品,置具塞锥形瓶中,加入70%(v/v)甲醇水溶液50.0 mL,密塞,超声提取30 min,放冷再次称重,用70%(v/v)甲醇水溶液补足失重,以12000 r/min的转速离心10 min,取上清液经0.22 μm微孔滤膜过滤,取续滤液500 μL,待测。

1.2.2 液相色谱条件

Achiral-chiral LC-MS/MS系统各模块连接示意图如图1所示。使用聚醚醚酮管将非手性柱和手性柱在线连接,利用Premixer Assy混合器将非手性柱洗脱液与左泵流动相充分混合。非手性色谱柱:Capcell core RP-C_18_色谱柱(150 mm×2.1 mm, 2.7 μm,日本Shiseido公司);手性色谱柱:AD-RH色谱柱(150×4.6 mm, 5.0 μm,日本Daicel公司)。右泵输送溶剂A(0.1%(v/v)甲酸水溶液)和溶剂B(乙腈),梯度洗脱程序为:0~4.0 min, 10%B; 4.0~15.0 min, 10%B~25%B; 15.0~23.0 min, 25%B~50%B; 23.0~28.0 min, 50%B~57%B; 28.0~34.0 min, 57%B~67%B; 34.0~44.0 min, 67%B~68%B; 44.0~56.0 min, 68%B; 56.0~60.0 min, 68%B~95%B; 60.0~60.1 min, 95%B~10%B; 60.1~66.0 min, 10%B;总流速为0.3 mL/min。左泵输送溶剂A和溶剂B,梯度洗脱程序为:0~4.0 min, 95%B~86%B; 4.0~15.0 min, 86%B~70%B; 15.0~23.0 min, 70%B~50%B; 23.0~28.0 min, 50%B~47%B; 28.0~34.0 min, 47%B~43%B; 34.0~44.0 min, 43%B~42%B; 44.0~56.0 min, 42%B; 56.0~60.0 min, 42%B~21%B; 60.0~60.1 min, 21%B~95%B; 60.1~66.0 min, 95%B;总流速为0.7 mL/min。进样体积为3 μL,色谱柱均置于35 ℃柱温箱中。

**图 1:**
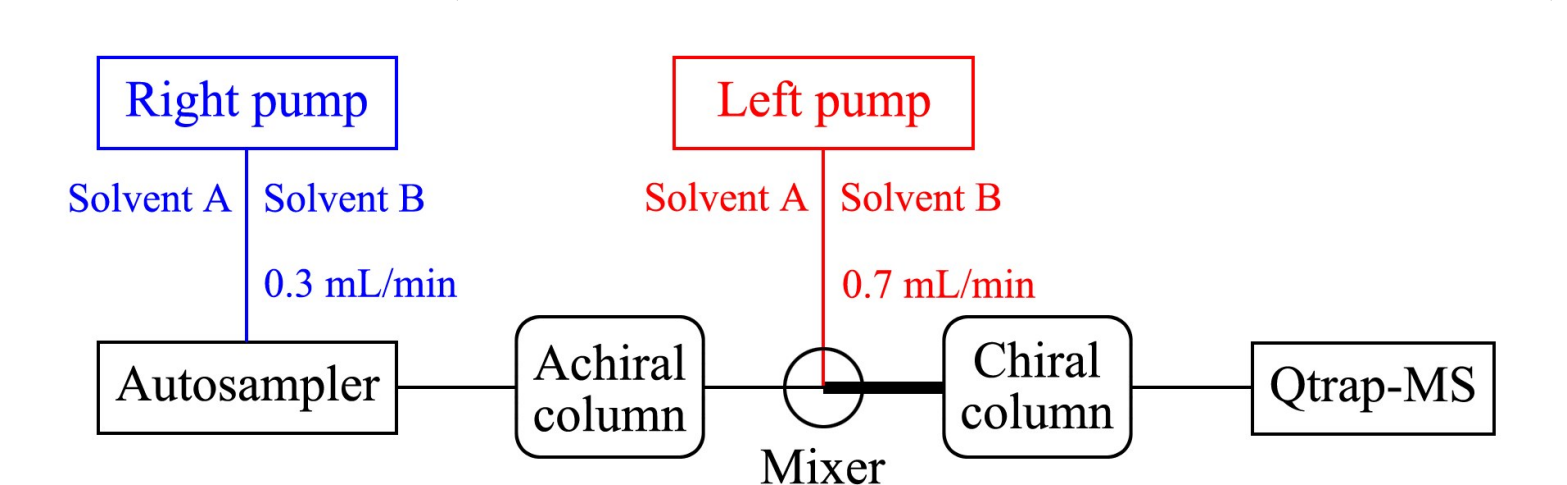
非手性-手性液相色谱-串联质谱系统构建示意图

1.2.3 质谱条件

手性色谱柱洗脱液直接导入三重四极杆质谱进行分析。电喷雾电离(ESI)源,正离子模式,预测多反应监测模式检测各化学成分。电喷雾电压:5500 V;离子化温度:550 ℃;雾化气压力(GS1): 379.2 kPa;辅助气压力(GS2): 379.2 kPa;气帘气压力(CUR): 241.3 kPa。Q1和Q3均为单位分辨率(0.6~0.8 Da)。预测离子对(Q1>Q3)及相关质谱参数(脱簇电压(DP)和碰撞能(CE))如表1所示,每个离子对的采集时间(dwell time)为10 ms。

**表 1:** 中药毛前胡化学成分在非手性-手性色谱-预测多反应监测系统上的质谱信息

No.	t_R_/ min	Molecular formula	Fragment ions (m/z)	Precursor ion (m/z)	Product ion (m/z)	Putative identity	DP/ V	CE/ eV	Chemical class	Ref.
1	2.92	C_5_H_9_NO_4_	129.60; 106.32; 84.04	148.06	84.04	glutamic acid	25	23	AA	[4]
2	3.00	C_10_H_13_N_5_O_5_	152.06	284.09	152.06	guanosine	50	25	AA	[4]
3	3.15	C_6_H_13_NO_2_	113.08; 86.08	132.10	86.08	isoleucine	50	18	AA	[4]
4	3.25	C_6_H_13_NO_2_	113.08; 86.08	132.10	86.08	leucine	40	30	AA	[4]
5	5.09	C_15_H_16_O_8_	163.04; 53.78	325.09	163.04	skimmin	80	25	SC	[3,4]
6	12.61	C_14_H_14_O_5_	245.08; 227.07; 191.03	263.09	245.08	trans-khellactone (TKL)	80	24	AP	[3]
7	13.89	C_14_H_14_O_5_	245.08; 191.04^*^	263.09	245.08	(3'S)-cis-khellactone (D-CKL)	80	27	AP	[3]
8	14.16	C_14_H_14_O_5_	245.08; 191.04^*^	263.09	245.08	(3'R)-cis-khellactone (L-CKL)	80	27	AP	[3]
9	15.84	C_20_H_24_O_9_	247.09; 229.08; 175.04	409.15	247.09	nodakenin	80	23	LF	[17,18]
10	19.50	C_14_H_14_O_5_	245.16^*^; 227.04; 203.06; 175.04	263.09	203.06	(D/L)-qianhucoumarin G (QC-G)	80	20	LF	[3,4]
11	19.70	C_14_H_14_O_5_	245.16^*^; 227.04; 203.06; 175.04	263.09	203.06	(D/L)-qianhucoumarin G	80	20	LF	[3,4]
12	22.31	C_13_H_10_O_5_	189.03	264.08	189.03	isopimpinellin	80	25	LF	[19]
13	23.91	C_21_H_24_O_9_	361.13; 245.08	438.17	245.08	3'-(hydroxyl-isovaleryl/2-methylbutyroyl)- 4'-hydroxyl-acetylkhellactone	80	30	AP	[3]
14	24.20	C_21_H_24_O_9_	361.14; 245.08; 187.03	438.17	245.08	3'-(hydroxyl-isovaleryl/2-methylbutyroyl)-4'-hydroxyl-acetylkhellactone	80	30	AP	[3]
15	24.22	C_16_H_16_O_6_	287.10; 245.08	305.10	245.08	qianhucoumarin B	80	30	AP	[4,20]
16	24.86	C_21_H_24_O_9_	361.14; 245.08; 187.03	438.17	245.08	3'-(hydroxyl-isovaleryl/2-methylbutyroyl)-4'-hydroxyl-acetylkhellactone	80	30	AP	[4]
17	25.73	C_12_H_8_O_4_	202.03; 174.06	217.04	202.03	bergapten or methoxsalen	80	20	LF	[21]
18	27.17	C_21_H_22_O_8_	343.12; 245.08	420.16	343.12	3'-(hydroxyl-tigloyl/senecioyl/angeloyl)- 4'-acetylkhellactone	80	15	AP	[3]
19	27.57	C_21_H_22_O_8_	343.11; 287.09; 245.08	420.16	343.11	3'-(hydroxyl-tigloyl/angeloyl/senecioyl)- 4'-acetylkhellactone	80	15	AP	[3]
20	29.29	C_16_H_14_O_5_	245.08; 217.08; 175.04	287.09	245.08	3'-acetyloxy-3',4'-dihydroseselin or 4'- acetyloxy-3',4'-dihydroseselin	80	35	AP	[3,4]
21	29.31	C_18_H_18_O_7_	287.09; 245.08; 227.07	369.09	245.08	qianhucoumarin D	80	32	AP	[4]
22	29.85	C_12_H_8_O_4_	202.03; 174.06; 165.72	217.04	202.03	bergapten or methoxsalen	80	20	LF	[21]
23	30.70	C_22_H_26_O_7_	329.14; 315.12; 245.08; 227.08	420.20	343.12	3'-(hydroxyl-tigloyl/angeloyl/senecioyl)- 4'-acetylkhellactone	80	15	AP	[4]
24	31.00	C_22_H_26_O_7_	329.14; 315.12; 245.08; 227.07	420.20	343.12	3'-(hydroxyl-tigloyl/angeloyl/senecioyl)- 4'-acetylkhellactone	80	15	AP	[4]
25	31.40	C_22_H_26_O_7_	329.14; 315.12; 245.08; 227.07	420.20	329.14	3'-(isovaleryl/2-methylbutyroyl)-4'-propi- onylkhellactone	80	30	AP	[3]
26	32.15	C_19_H_20_O_7_	287.09; 245.08	378.15	245.08	3'-acetyl-4'-propionylkhellactone	80	30	AP	[3]
27	32.34	C_19_H_20_O_7_	287.09; 245.08	378.15	245.08	3'-acetyl-4'-propionylkhellactone isomer	80	30	AP	[3]
28	33.52	C_19_H_20_O_6_	245.08; 217.07; 205.04; 191.04	345.13	245.08	Pd-C-I	80	30	LP	[17]
29	35.18	C_20_H_22_O_7_	287.09; 245.08	392.17	245.08	bocconin	80	15	AP	[3,4]
30	35.61	C_19_H_20_O_6_	345.13; 245.08; 261.05	362.15	261.05	3'-(isovaleryloxy/2-methylbutyroyloxy)- 4'-oxo-3',4'-dihydroseselin	80	30	AP	[3]
31	35.64	C_20_H_22_O_7_	287.09; 245.08	392.17	245.08	isobocconin	80	15	AP	[4]
32	36.19	C_19_H_20_O_6_	345.13; 261.06; 245.08	362.15	261.06	3'-(isovaleryloxy/2-methylbutyroyloxy)- 4'-oxo-3',4'-dihydroseselin	80	30	AP	[3]
33	36.27	C_11_H_6_O_4_	175.04; 147.05	203.03	147.05	xanthotoxol	80	40	LF	[19]
34	36.45	C_16_H_14_O_4_	203.03; 175.04; 159.03; 147.04; 131.02	271.09	203.03	imperatorin	80	18	LF	[3,19]
35	37.69	C_21_H_22_O_7_	327.12; 287.09; 245.08; 227.07^*^	409.12	245.08	(3'S)-pteryxin (D-Pte)	80	20	AP	[3,4]
36	37.71	C_16_H_14_O_5_	245.08; 217.09; 175.04	287.09	245.08	4'-acetyl-khellactone	80	32	AP	[3,22]
37	38.04	C_21_H_22_O_7_	327.12; 287.09; 245.08^*^; 227.07	409.12	227.07	(3'S)-praeruptorin A (D-PA)	80	19	AP	[3,22]
38	38.12	C_24_H_28_O_7_	349.10; 327.12; 251.06; 245.08; 227.07	451.17	245.08	3'-(isovaleryl/2-methylbutyroyl)-4'-(tigloyl/ senecioyl/angeloyl)-khellactone	80	34	AP	[4]
39	38.50	C_17_H_16_O_5_	283.08; 233.04; 218.05; 189.12; 173.03	301.10	233.04	cnidilin	80	37	LF	[4]
40	38.94	C_16_H_14_O_5_	245.08; 175.04	287.09	245.08	3'-acety-4'-dehydro-decusinol	80	32	LP	-
41	39.01	C_23_H_28_O_7_	351.12; 245.08; 227.07	434.21	245.08	3'-(2-isobutyroyl)-4'-(isovaleryl/2-meth- ylbutyroyl)-khellactone	80	30	AP	[4]
42	39.03	C_21_H_24_O_7_	329.13^*^; 287.09; 245.08	406.18	245.08	(3'S)-3'-isovaleryl-4'-acetylkhellactone (D-IAK)	80	30	AP	[3]
43	39.49	C_21_H_22_O_7_	327.12; 287.09; 245.07; 227.07^*^	409.12	245.08	(3'R)-pteryxin (L-Pte)	80	20	AP	[3,4]
44	39.58	C_21_H_22_O_7_	327.12; 287.09; 245.08^*^; 227.07	409.12	227.07	(3'R)-praeruptorin A (L-PA)	80	19	AP	[3]
45	41.42	C_16_H_14_O_4_	203.05; 175.04; 159.03; 147.03; 131.02	271.06	203.05	isoimperatorin	80	18	LF	[3,19]
46	41.60	C_21_H_24_O_7_	329.13^*^; 287.09; 245.08	406.18	245.08	(3'R)-3'-isovaleryl-4'-acetylkhellactone (L-IAK)	80	30	AP	[3]
47	45.84	C_23_H_26_O_7_	315.12; 245.08	432.20	315.12	3'-(2-isobutyroyl)-4'-(tigloyl/senecioyl/angeloyl)-khellactone	80	15	AP	[3,4]
48	46.05	C_23_H_26_O_7_	327.13; 315.12; 227.09	432.20	227.09	3'-(2-isobutyroyl)-4'-(tigloyl/senecioyl/ angeloyl)-khellactone	80	30	AP	[3,4]
49	47.41	C_24_H_26_O_7_	327.12; 227.07	444.20	327.12	3'-(tigloyl/senecioyl/angeloyl)-4'-(tigloyl/ senecioyl/angeloyl)-khellactone	80	25	AP	[3,4
50	48.16	C_24_H_26_O_7_	327.12; 245.08; 217.08; 189.02	444.20	327.12	3'-(tigloyl/senecioyl/angeloyl)-4'-(tigloyl/ senecioyl/angeloyl)-khellactone	80	26	AP	[3,4]
51	48.90	C_23_H_28_O_7_	351.13; 245.08; 227.07	434.21	245.08	3'-(2-isobutyroyl)-4'-(isovaleryl/2-meth- ylbutyroyl)-khellactone	80	30	AP	[3]
52	49.65	C_24_H_26_O_7_	349.11; 327.12; 245.08^*^; 227.06	444.20	327.12	(3'S)-praeruptorin B(D-PB)	80	36	AP	[3,4,22]
53	50.28	C_24_H_28_O_7_	329.14; 327.12; 227.06;	451.17	245.08	3'-(isovaleryl/2-methylbutyroyl)-4'-(tigloyl/ senecioyl/angeloyl)-khellactone	80	34	AP	[3,4]
54	50.50	C_24_H_26_O_7_	349.11; 327.12; 245.08^*^; 227.06	444.20	327.12	(3'R)-praeruptorin B (L-PB)	80	36	AP	[3,4,22]
55	51.61	C_24_H_28_O_7_	349.11; 327.11; 251.06; 245.08; 227.07^*^	451.17	245.08	(3'S)-praeruptorin E (D-PE)	80	35	AP	[3,4]
56	54.31	C_24_H_28_O_7_	349.11; 327.11; 251.06; 245.08; 227.07^*^	451.17	245.08	(3'R)-praeruptorin E (L-PE)	80	35	AP	[3,4]
57	55.08	C_24_H_30_O_7_	329.13; 245.08^*^; 227.06	448.23	227.06	(3'S)-3',4'-diisovalerylkhellactone (D-DIK)	80	55	AP	[3,4]
58	56.49	C_24_H_30_O_7_	329.13; 245.08^*^; 227.06	448.23	227.06	(3'R)-3',4'-diisovalerylkhellactone (L-DIK)	80	55	AP	[3,4]
59	63.18	C_24_H_30_O_7_	329.13; 245.08; 227.06	448.23	227.06	3',4'-diisovalerylkhellactone isomer	80	55	AP	
60	64.75	C_24_H_26_O_7_	327.12; 245.08; 217.08; 189.02	444.20	327.12	3'-(tigloyl/senecioyl/angeloyl)-4'-(tigloyl/ senecioyl/angeloyl)-khellactone	80	36	AP	[3,4]

DP: declustering potential; CE: collision energy; * quantifier ion; Pd-C-I: peucedanum decursivum coumarin Ⅰ; AA: amino acid; SC: simple coumarin; AP: angular-type pyranocoumarin; LF: linear-type furocoumarin; LP: linear-type pyranocoumarin; -: new compound.

由于对映异构体质谱行为完全一致,因此本研究对于每个化合物均采用两对离子对进行检测,并利用定量和定性离子对丰度比值(QQR)判定两个色谱信号是否为对映异构体^[23,24]^。利用数据依赖性采集模式触发增强子离子扫描(EPI)模式记录各母离子的MS^2^图谱。EPI实验参数为:CE, 40 eV;碰撞能量分散(CES), 35 eV。利用Analyst 1.6.2软件对数据进行分析。

## 2 结果与讨论

### 2.1 液相条件的优化

课题组前期在建立反相色谱和亲水作用色谱直接耦联系统^[25,26]^时发现,通过选择内径小、柱效高的反相色谱柱作为前端色谱柱可以达到较好的分离效果。本实验筛选并考察了多个候选色谱柱,如ACE UltraCore 2.5 Super C_18_ (150 mm×2.1 mm, 2.5 μm,英国Advance Chromatography Technologies公司)、Waters Acquity UPLC HSS T3 (100 mm×2.1 mm, 1.8 μm,美国Waters公司)、Ascentis^®^ Express F5 (150 mm×3.0 mm, 2.7 μm,美国Sigma-Aldrich公司)、Capcell core RP-C_18_(150 mm×2.1 mm, 2.7 μm,日本Shiseido公司)等多款核壳型或新型填料高效色谱柱,以色谱峰形、分离度作为指标衡量色谱柱的分离效能,最终选择Capcell core RP-C_18_色谱柱作为前端化学选择性分离柱。

进一步优化非手性柱流动相流速(0.15、0.20、0.30和0.35 mL/min)和手性柱流动相流速(0.60、0.70和0.80 mL/min)、流动相的洗脱程序以及在水中加入甲酸的体积分数(0%、0.05%、0.10%和0.15%)。最终选定0.1%甲酸作为水相的添加剂;非手性柱和手性柱的流速分别为0.3 mL/min和0.7 mL/min;具体洗脱程序见1.2.2节。

前期研究表明,AD-RH色谱柱^[3,7,27]^对APs对映异构体有较好的分离效果,其反相分离机制与前端色谱柱能够很好地兼容。在APs中,C-3'和C-4'取代基团的大小会影响对映异构体的分离效能,且取代基团越大,分离效果越差,需要采用较低比例的有机相和较长的洗脱时间才能达到较好的分离。因此,在洗脱过程中逐步降低流动相的比例,且在RP-C_18_和AD-RH色谱柱之间引入稀释泵,可以对含有大取代基团的对映异构体实现良好的色谱分离。对映异构体在AD-RH手性色谱柱上的流出顺序决定于手性中心与表面涂敷有直链淀粉-三(3,5-二甲基苯基氨基甲酸酯)的硅胶之间的作用力^[28]^。课题组前期研究^[3]^发现,(3*'S*,4*'S*)构型的对映异构体与固定相的作用力小于其(3*'R*,4*'R*)对映异构体,其被率先洗脱出来。因此,毛前胡中具有较小保留时间的对映异构体构型被认为是(3*'S*,4*'S*)构型,为了表述方便,以下简称为3*'S*构型。

### 2.2 多反应监测模式的条件优化

结合已经报道^[29]^的质谱裂解规律及MRM离子对信息,笔者首先对预测离子对的化学结构进行验证,进而采用预测多反应监测模式检测各化学成分,并利用EPI模式获取化合物的二级质谱信息,结合碎片离子对化合物进行鉴定。前期研究^[2]^表明,毛前胡中所含的化学成分与前胡相似,不同成分的含量差异大,其中含量较高的APs可能会影响微量化合物的检出。因此,本实验引入在线能量分辨质谱法^[30,31]^对各化合物的碰撞能进行优化,采用非最优碰撞能抑制高含量成分的质谱响应,从而实现所有化合物的检出。

### 2.3 对映体异构体判别

实验通过QQR判定是否为对映异构体^[23,24]^,结合对映异构体在achiral-chiral LC系统上的保留时间大于在单柱RP-C_18_色谱柱上的保留时间,经过验证,发现顺式-凯琳内酯(CKL, 7和8(表1中化合物的顺序编号))、前胡香豆素G(QC-G, 10和11)、顺式-北美芹素(Pte, 35和43)、白花前胡甲素(PA, 37和44)、顺式-3'-异戊酰-4'-乙酰凯琳内酯(IAK, 42和46)、白花前胡乙素(PB, 52和54)、白花前胡素E (PE, 55和56)和顺式-3',4'-双异戊酰凯琳内酯(DIK, 57和58)共计8对对映异构体,所有对映异构体在非手性-手性色谱耦联系统上均能较好地手性分离(见图2),除前胡香豆素G为LFs外,其余均为APs。如化合物顺式-北美芹素(35和43)为CKL的衍生物,在角型吡喃香豆素母核的C-3'和C-4'分别为乙酰基和当归酰基取代,分子式为C_21_H_22_O_7_,预测离子对包括*m/z* 409.12>327.12、409.12>287.09、409.12>245.07、409.12>227.07,利用数据依赖性采集模式触发EPI,获取母离子的二级质谱信息,验证化合物的结构。选择*m/z* 409.12>227.07和409.12>245.07作为定量离子对和定性离子对,化合物L-Pte和D-Pte的QQR均为2.36,根据两者的保留时间,鉴定L-Pte和D-Pte分别为3'*S*和3*'R*构型。化合物顺式-北美芹素的叠加选择离子流图和二级质谱图如图3a和3b所示。

**图 2:**
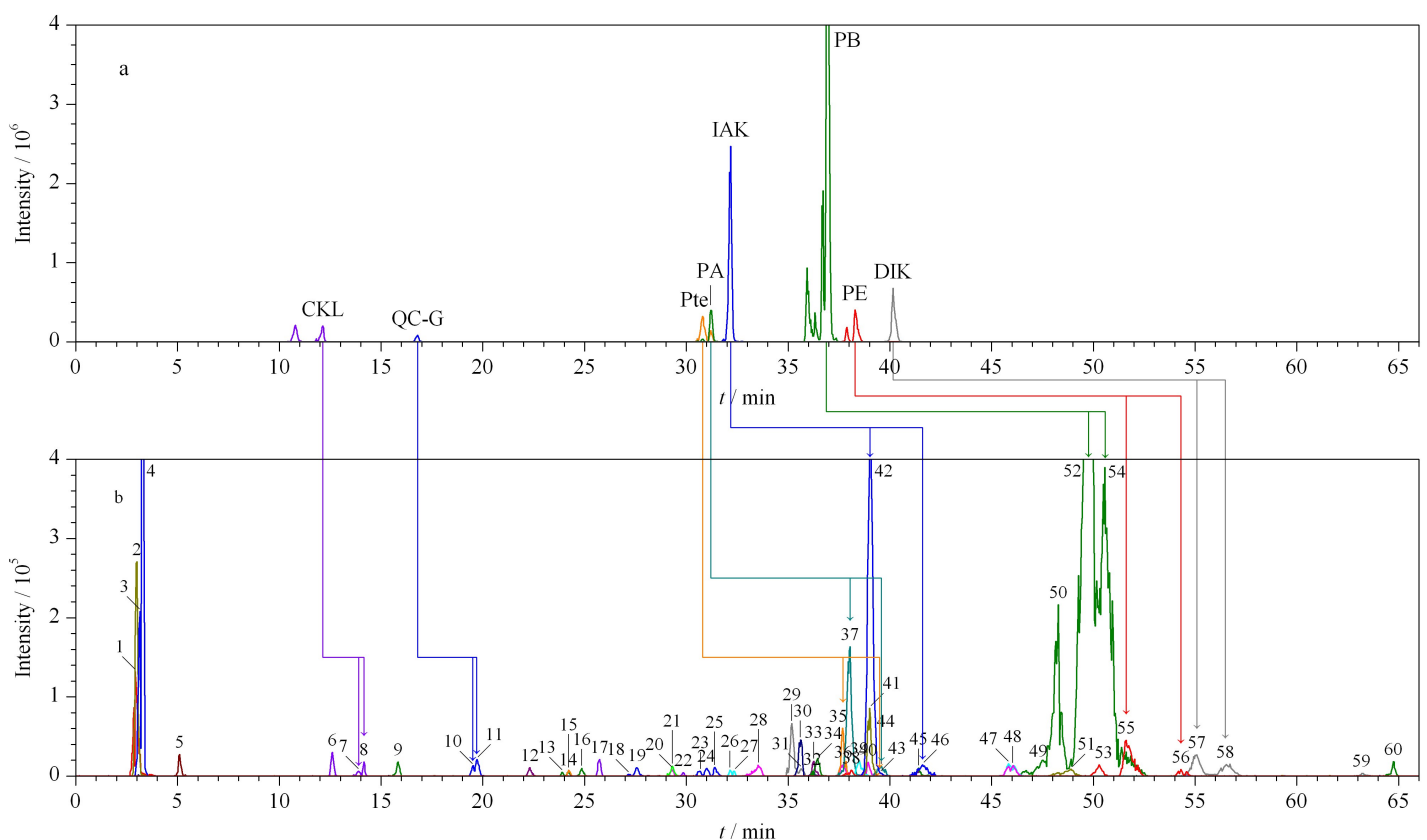
采用(a)非手性色谱-串联质谱和(b)非手性-手性色谱-串联质谱时毛前胡的选择离子流色谱图

**图 3:**
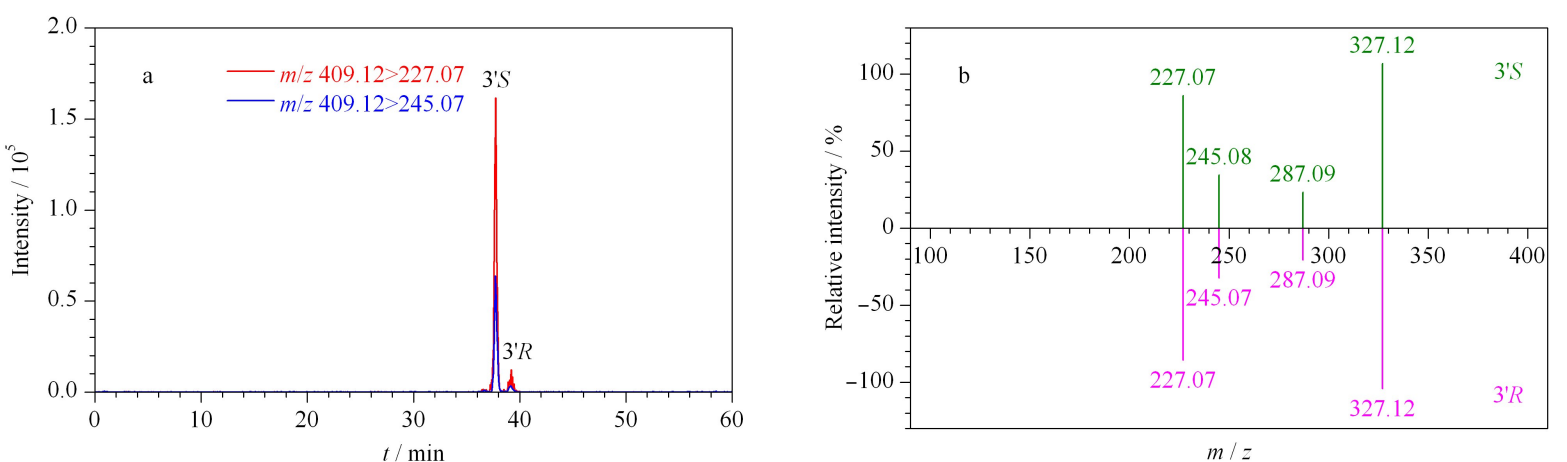
顺式-北美芹素的(a)叠加选择离子流色谱图和(b)二级质谱图

### 2.4 毛前胡结构鉴定及质谱裂解规律

2.4.1 化学成分定性分析

利用建立的非手性-手性色谱-预测多反应监测系统采集毛前胡在正离子模式下的质谱信息,结合碎片离子信息、文献对比、数据库搜索以及质谱裂解规律推导,对所有响应值大于1000 cps,且信噪比(*S/N*)大于100的信号进行结构确认,初步鉴定了其中60个化学成分,包括4个AAs、43个APs、10个LFs、2个LPs以及1个SC化合物。

各化合物的保留时间、主要碎片离子、预测离子对以及可能的化学成分见表1。

在正离子模式下,香豆素类化合物易产生准分子离子峰[M+H]^+^或加合离子峰[M+Na]^+^及[M+NH_4_]^+^。APs主要为顺式凯琳内酯的衍生物,大部分APs通过C-3'和C-4'的羟基结合不同的取代基形成不同的化合物,常见的取代基有乙酰基、当归酰基、巴豆酰基、千里光酰基、正丁酰基、异戊酰基等。对于LFs来说,主要通过C-5和C-8或C-3'和C-4'上取代基的不同,形成不同的化合物,如异戊烷氧基、甲氧基、糖基等。以下对毛前胡中的主要化学类型的质谱裂解规律以及各化学成分的鉴定过程进行分述。

2.4.2 角型吡喃香豆素类

从毛前胡提取物中共鉴定出43个APs成分,大部分为顺式凯琳内酯的衍生物。对于APs来说,C-4'取代基处于香豆素母核的苄位,可与香豆素母核形成大的共轭体系,得到更稳定的子离子。通过丢失C-3'和C-4'位的取代基,形成*m/z* 245.07特征性碎片离子,并进一步通过中性丢失水分子形成另一个*m/z* 227.07特征性碎片离子。例如,PA在C-3'和C-4'位置分别有一个当归酰基和乙酰氧基取代,分子式为C_21_H_22_O_7_,最显著的准分子离子峰为*m/z* 409.12 [M+Na]^+^。化合物裂解时,首先从C-4'中性丢失一分子乙酰氧基(60 Da),形成丰度最高的碎片离子*m/z* 327.12 [M+H-CH_3_COONa]^+^、进而丢失C-3'取代基(C_4_H_6_CO, 82 Da),形成特征碎片离子*m/z* 245.08 [M+H-CH_3_COOH-C_4_H_6_-CO]^+^,随后进一步中性丢失一分子水,最终形成*m/z* 227.07 [M+H-CH_3_COOH-C_4_H_7_COOH]^+^的特征碎片离子。PA的质谱图和可能的裂解途径如图4所示。

**图 4:**
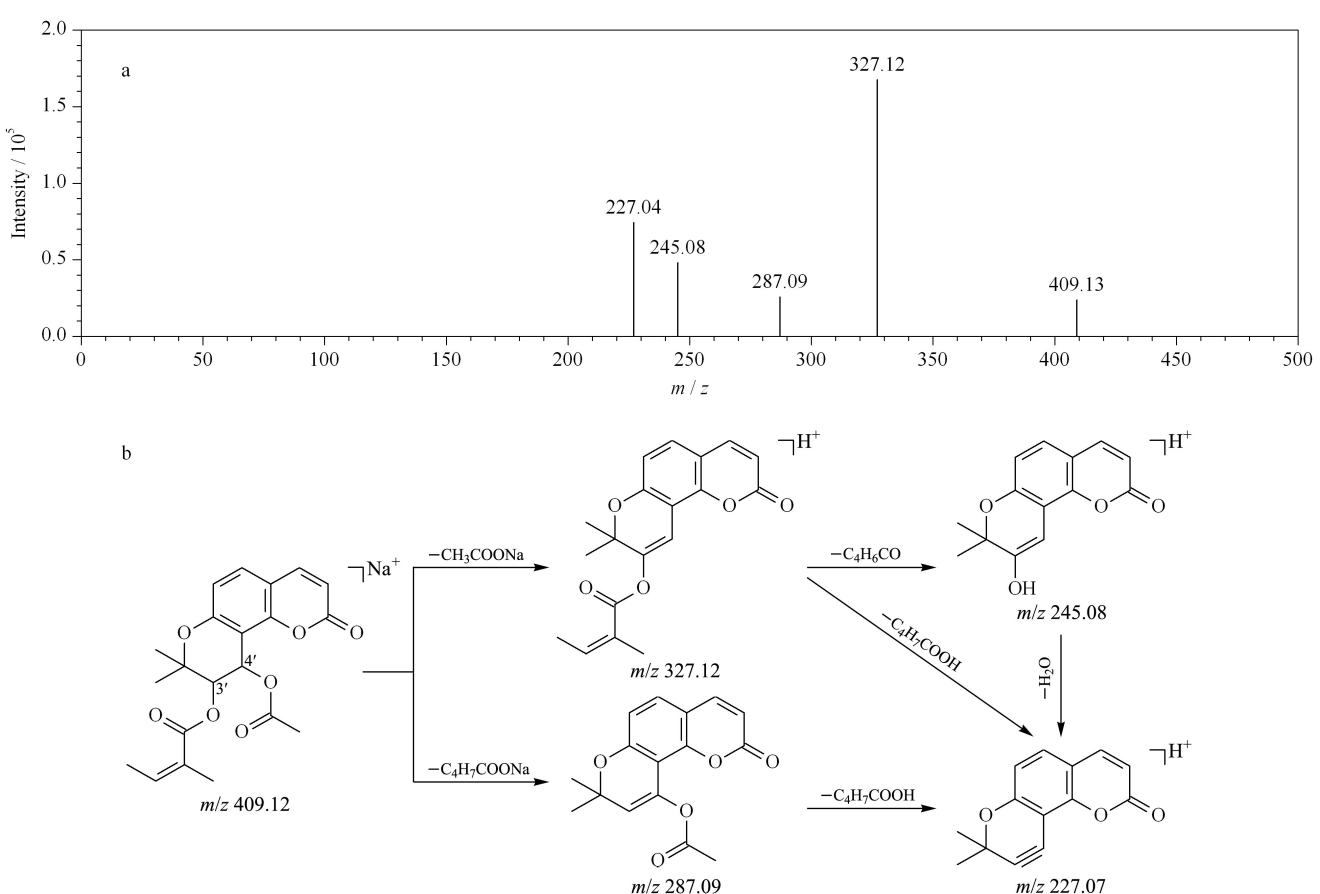
白花前胡甲素的(a)质谱图和(b)可能的裂解途径

2.4.3 线型呋喃香豆素类

除APs外,毛前胡中还含有少量的LFs化学成分,其中包括一对对映异构体QC-G (10和11)。质谱裂解规律主要由C-5、C-8或C-3'、C-4'取代基的不同而产生,常见的取代基有异戊烷氧基、甲氧基、羟基、糖基等。

C-5或C-8取代基首先通过中性丢失形成*m/z* 203.05碎片离子,随后进一步中性丢失CO (28 Da)和CO_2_(44 Da),分别产生*m/z* 175.04和159.03的特征性碎片离子^[21]^。C-3'或C-4'取代的化合物,通过中性丢失C-3'或C-4'上的取代基,形成*m/z* 247.08和229.20的特征性碎片离子^[19]^。如紫花前胡苷,准分子离子峰*m/z* 409.08 [M+H]^+^,分子式为C_20_H_24_O_9_, C-3'有葡萄糖基取代,中性丢失一分子葡萄糖残基,产生*m/z* 247.08 [M+H-C_6_H_10_O_5_]^+^的碎片离子,随后,进一步丢失一分子水,形成*m/z* 229.20 [M+H-C_6_H_10_O_5_-H_2_O]^+^的特征性离子。紫花前胡苷质谱图和可能的裂解途径如图5所示。

**图 5:**
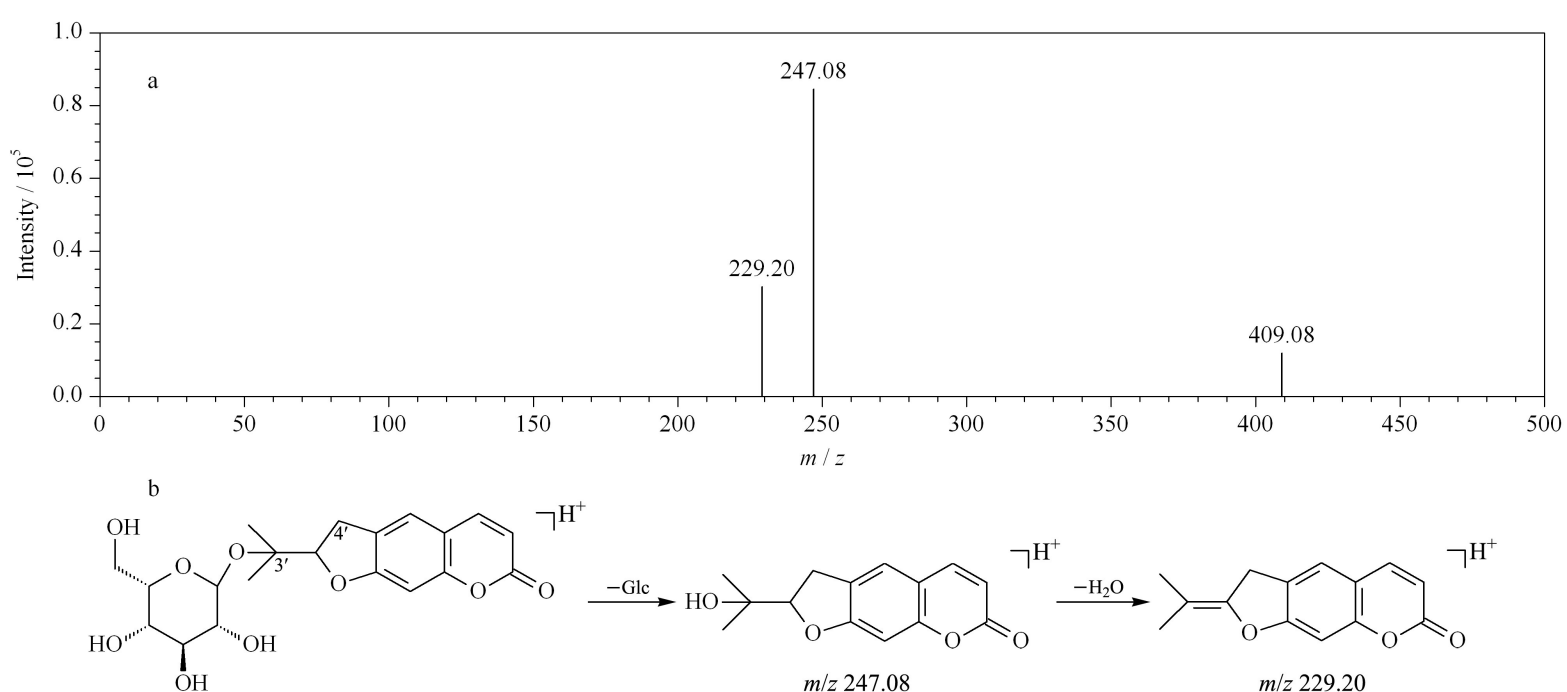
紫花前胡苷的(a)质谱图和(b)可能的裂解途径

### 2.5 非手性-手性色谱-预测多反应监测法优缺点

非手性-手性色谱耦联系统结合了RP-C_18_的高效分离能力以及手性色谱柱的立体选择性优势,可以同步实现化合物的化学选择性和立体选择性分离。相比较于中心切割非手性-手性二维液相色谱而言,非手性-手性色谱耦联系统具有仪器配置简单、重复性好、精密度高的优点。然而,该系统也存在一定的缺点,如分离机制复杂,因为每个化合物都要经历2个色谱柱;寻找最佳的洗脱条件是一个具有挑战性的任务,因为输送溶剂最少需要4个泵;二维色谱柱的要求高,要兼顾一维色谱柱的洗脱能力等。

## 3 结论

中药中广泛存在对映异构体,这些对映异构体往往具有不同的生物活性,从而发挥不同的治疗效果。全面阐明中药的化学成分组成,深入揭示对映异构体的含量差别将会使药物更好地发挥药理活性。本研究建立了非手性-手性色谱-预测多反应监测法,实现了中药毛前胡的化学成分解析,揭示了其对映异构体的含量差异。本研究将为含有对映异构体的中药及其他复杂样品的深入定性、定量分析提供可靠的方法。
